# Strong HLA-DR expression in large bowel carcinomas is associated with good prognosis.

**DOI:** 10.1038/bjc.1993.290

**Published:** 1993-07

**Authors:** S. N. Andersen, T. O. Rognum, E. Lund, G. I. Meling, S. Hauge

**Affiliations:** Institute of Forensic Medicine, National Hospital, University of Oslo, Norway.

## Abstract

**Images:**


Br. J. Cancer (1993), 68, 80-85 © Macmillan Press Ltd., 1993

### Strong HLA-DR expression in large bowel carcinomas is associated with good prognosis

S. Norheim Andersen[1], T.O. Rognum[1], E. Lund[2], G.I. Meling[1] & S. Hauge[1]

[1]*Institute of Forensic Medicine, The National Hospital, University of Oslo, and* [2]*Institute of Community Medicine, University of Tromsø, Norway.*

**Summary** One hundred large bowel carcinomas operated on between 1978 and 1982 were studied immunohistochemically with regard to expression of HLA-DR antigens. Three sections from each tumour were investigated by a semiquantitative scoring system, and a mean score for each patient established. Based on this scoring system, the tumours were divided into three groups: 0; 0.1-1.0; and > 1.0. All patients were followed until death ($n = 68$) or until June 1, 1992, and all cancer-specific deaths ($n = 56$) have been recorded. Analysis of survival in the whole patient group showed significant difference between the three levels of tumour HLA-DR expression ($P = 0.006$); patients who had tumours with strong HLA-DR expression showing the best survival. In a stratified analysis after Dukes' stages there was still a significant difference ($P > 0.001$) between the three levels of HLA-DR staining intensity. After a multiple regression analysis (Cox) with correction for different variables, the HLA-DR expression maintained its significance as a risk factor. To our knowledge this is the first time a relationship between intensity of tumour DR expression and survival has been shown in large bowel carcinoma.

Several attempts have been made to define histopathological characteristics of prognostic significance for patients with large bowel carcinoma. A positive correlation between histological tumour grade and survival time has been reported (Remmele & Heine, 1981), but up to now, staging has been considered the most reliable way to assess prognosis (Dukes & Bussey, 1958; Turnbull *et al.*, 1967; Remmele & Heine, 1981). The preoperative plasma CEA level has proved valuable in follow-up studies after surgery for large bowel carcinomas (Wanebo *et al.*, 1978; Rognum, 1986; Meling *et al.*, 1992). During the last years several papers applying flow cytometric DNA determination methods in human solid tumours have been published, and studies have shown that the presence of distinctly aneuploid DNA ploidy pattern in large bowel carcinoma worsens survival rates significantly (Wolley *et al.*, 1982; Rognum *et al.*, 1987*a*, 1991).

HLA class II antigen expression has recently been related to good prognosis in squamous cell carcinoma of the larynx (Esteban *et al.*, 1990) and breast carcinoma (Concha *et al.*, 1991), whereas HLA-DR positivity was related to a bad prognosis in malignant melanoma (Zaloudik *et al.*, 1988). In large bowel carcinomas the association between HLA-DR expression and prognosis is less clear, since Gutierrez *et al.* (1987) found a correlation between DR-expression and prognosis according to Jass's stages (Jass *et al.*, 1986), while Möller *et al.* (1991) did not find any influence of HLA-DR on disease-free survival.

The purpose of the present study was to test a possible prognostic significance of tumour DR-expression in 100 large bowel carcinomas with at least 10 years postoperative observation time.

### Patients, materials and methods

### *Patients*

One hundred large bowel carcinomas from 100 patients operated on between 1978 and 1982 were studied immunohistochemically with regard to expression of HLA-DR antigens. The mean age of the patients was 64.5 years (range 28 to 89 years), and there were 52 men and 48 women. All tumours were staged according to the extended Dukes' scheme, which in addition to Dukes' stages A, B, and C, ascribed stage D to tumours with distant organ metastases and inoperable tumours (Turnbull *et al.*, 1967). The tumours were in addition graded as well-, moderately-, or poorly differentiated (Ashley, 1978). Gross pathological examination was performed by the same observer (T.O.R.) throughout the study. The distribution of the variables is given in Table I.

### *Immunohistochemistry*

Tissue slices from each tumour were fixed in cold 96% ethanol and processed for paraffin embedding as described previously (Brandtzaeg, 1974). Sections cut at 6 µm were dewaxed and subjected to immunofluorescence staining at room temperature. One section from each series was stained by a trichrome routine method (HAS) containing haematoxylin, azofloxin and saffron (Stave & Brantzaeg, 1977).

A murine monoclonal antibody to a nonpolymorphic human HLA-DR antigen (Beckton Dickinson, Sunnyvale, Calif., USA) was applied (1:20 for 20 h) in an indirect 3-step immunofluorescence method (Brandtzaeg & Rognum, 1983), including affinity purified biotinylated horse anti-mouse IgG (0.05 g IgGl$^{-1}$, 3 h) and fluorescein isothiocyanate (FITC)-labelled avidin (0.05 gl$^{-1}$, 30 min), both purchased from Vector Laboratories (Burlingame, Calif., USA). The horse reagent was absorbed with normal human serum to avoid interspecies cross-reactions.

To test the specificity of the staining procedure, the primary antibody was omitted in adjacent sections from a DR-positive tumour.

To test the specificity of the primary antibody, an adjacent section from a DR-positive tumour was stained with a monoclonal antibody of the same isotype, but without specificity relevant for colon tissue (monoclonal antibody against a neoepitope on the complement factor C9; Mollnes *et al.*, 1985).

Observations were done in a Leitz Aristoplan fluorescence microscope equipped with an Osram Hg 100 W lamp for fluorescein (green emission). Narrowband excitation and selective filtration of the fluorescence colour were obtained with a Ploem-type epi-illuminator. In 97 of the 100 tumours, three different blocks of tumour tissue were evaluated – one from the centre of the tumour and two from the periphery – while in the last three tumours we only had 2, 2 and 1 block, respectively. Both the tumour and the transitional mucosa were evaluated. The epithelial staining for HLA-DR antigens were scored semiquantitatively on arbitrary scales from 0 to 3. HLA-DR+ cells showed usually diffuse distribution of such determinants throughout the cytoplasm with peripheral

Correspondence: S. Norheim Andersen, Institute of Forensic Medicine, The National Hospital, University of Oslo, 0027 Oslo, Norway.
Received 14 October 1992; and in revised form 2 February 1993.

**Table I** Clinicopathologic characteristics of the patients

| | |
|---|---|
| Number of patients | 100 |
| Sex | |
| Male | 52 |
| Female | 48 |
| Mean age (yr) | 64.5 |
| Range | 28–89 |
| Mean follow-up (yr) | 11.4 |
| Range | 10–14.3 |
| Dukes' stage | |
| A | 20 |
| B | 36 |
| C | 25 |
| D | 19 |
| Histological grade | |
| Well differentiated | 11 |
| Moderately differentiated | 70 |
| Poorly differentiated | 19 |
| Localisation | |
| Right colon | 32 |
| Left colon | 25 |
| Rectum | 43 |

intensification, particularly apically in glandular structures. Both staining intensity and distribution area of staining were taken into account, each contributing about 50% of the sum. A score of 0 was given for virtually no staining: and 3 for intense overall fluorescence. 'Tumour score' was the mean score of all blocks from each tumour. The same investigator (S.N.A.) was responsible for the fluorescence scoring throughout the study; a blind test for both intra- and inter-observer reproducibility was performed. For the inter-observer test new sections were cut and immunostained. Proportions of agreement (P.) and Kappa values were estimated in the analysis of observer variability (Landis & Koch, 1977).

The distribution of the DR was, in addition, separately categorised as heterogenous (extensive, intermediate or slight) or homogenous. An extensive degree of heterogenous staining refers to the presence of abrupt transition between positive and negative epithelium within the same crypt (Rognum *et al.*, 1983).

**Figure 1** Moderately differentiated large bowel carcinoma. **a**, HAS staining (magnification × 20). **b**, Adjacent section stained green for HLA-DR determinants (magnification × 80).

*Calculation of survival rates*

All patients were followed until death or until June 1, 1992, with a mean of 11.4 years (range 10 to 14.3 years). Survival rates were computed by the actuarial or life-table method. Crude survival was based on all deaths; for corrected survival rates, deaths not due to cancer were censored at time of death. Information about recurrences and deaths due to cancer was obtained from the hospital records or the post-mortem reports. The log-rank procedure was used to assess the statistical significance between survival distribution.

To estimate the effect of different factors on survival, a multivariate analysis was done by the proportional-hazards method proposed by Cox (1972). Included in the analysis were the following factors: age, sex, clinicopathological stage, histologic grade, DNA ploidy pattern, DR staining, preoperative CEA plasma level, and tumour site.

### Results

*HLA-DR staining pattern, Dukes' stage, and histological grade*

When omitting the primary antibody or replacing it with an antibody of the same isotype (IgG2a), no staining was seen in adjacent sections from a tumour which stained positive with the anti-HLA-DR antibody.

Based on three samples, 52 tumours showed HLA-DR positivity in varying degrees (Figure 1) and 48 were negative. There was some degree of intratumour variation; when evaluating only one section from each specimen, 38 were positive and 62 negative. Consequently, a mean score for each tumour was based upon the average of the scores from the three sections. The tumours were then divided into three groups according to the mean score: 0; 0.1–1.0; and >1.0.

There were no significant differences in HLA-DR score between tumours in each Dukes' stage (Table II), whereas there were slightly more DR-positive tumours in the poorly differentiated group, compared to the better differentiated tumours ($P = 0.04$), (Table III).

Concerning the heterogeneity of HLA-DR staining in the tumour, 39 of the tumours showed variable staining throughout the tumour tissue, whereas 61 showed a homogenous staining pattern, either positive (13) or negative (48). No difference in degree of heterogeneity was observed with regard to Dukes' stage, while there was a tendency for poorly differentiated carcinomas to be more homogenously stained ($P = 0.04$).

Intraobserver reproducibility was 'substantial' (Kappa = 0.61) and interobserver (including repeated sectioning and staining) reproducibility was 'moderate' (Kappa = 0.46) (Figure 2), according to the classification by Landis and Koch (1977).

*Tumour HLA-DR expression and survival analysis*

During the follow-up period there were 56 cancer specific deaths, whereas 12 patients died from other causes. Analysis of survival in the whole patient group showed significant differences between the three levels of tumour HLA-DR expression: 0; 0.1–1.0; >1.0 ($\chi^2 = 10.214$, d.f. = 2, $P = 0.006$), (Figure 3). In a stratified analysis after Dukes' stages, there was still a significant difference ($P < 0.001$) between the three levels of HLA-DR staining intensity (Figure 4).

Only one patient with tumour score for HLA-DR above 1.6 died from his cancer. This 73 year-old male patient, operated on for a rectal carcinoma, Dukes' stage B, survived for 6.8 years. Another male patient with a poorly differentiated Dukes'stage D rectal tumour with a HLA-DR score of 1.5, survived for 2.8 years.

According to the multivariate analysis, adjustment for other risk factors did not essentially change the estimate of the prognostic effect of HLA-DR expression on the relative risk for death from large bowel carcinoma (Table IV).

**Table II** HLA-DR expression according to Dukes' stages in colorectal carcinoma patients

| *Dukes' stage* | *HLA-DR expression* 0 | *0.1–1.0* | *>1.0* | *Total* |
|---|---|---|---|---|
| A | 9 | 10 | 1 | 20 |
| B | 17 | 13 | 5 | 35 |
| C | 8 | 13 | 5 | 26 |
| D | 14 | 4 | 1 | 19 |
| Total | 48 | 40 | 12 | 100 |

Chi square = 10.01; d.f. 6; $P = 0.12$.

**Table III** HLA-DR expression according to histological grade

| *Histological grade* | *HLA-DR expression* 0 | *0.1–1.0* | *>1.0* | *Total* |
|---|---|---|---|---|
| Well | 5 | 6 | 0 | 11 |
| Moderately | 35 | 29 | 6 | 70 |
| Poorly | 8 | 5 | 6 | 19 |
| Total | 48 | 40 | 12 | 100 |

Chi square = 9.79; d.f. 4; $P = 0.04$.

**Figure 2** Scatter diagrams of interobserver and intra-observer reproducibility. The degree of accordance for HLA-DR score **a**, between the first observation by SNA and the second one by TOR after new slicing and staining ($P_o = 0.63$ (17/27) Kappa 0.46), and **b**, between two observations by the same observer (SNA), the last performed blindly after 12 weeks ($P_o = 0.7$ (14/20) Kappa 0.61).

### Discussion

The main finding of the present paper was the significant relationship between the HLA-DR expression of the tumour cells and survival of the patients. To our knowledge this is the first report of such a relationship, and it was surprising, since we (like Ghosh *et al.*, 1986), were unable to demonstrate association between HLA-DR expression and Dukes' stage.

**Figure 3** Survival curves (corrected for deaths not due to cancer) for patients with different degrees of HLA-DR expression, as evaluated by immunohistochemical scoring. The patients are divided into three groups, according to the staining intensity. Interval of the semiquantitative scores are given by different types of lines in the figure; patients whose tumours have no HLA-DR expression (——); weak DR expression, 0.1–1.0 (.....); and strong DR expression, >1.0 (---). The difference between the three levels is statistically significant ($P = 0.006$).

Under normal conditions colonic mucosa does not express HLA class II molecules (Daar *et al.*, 1982; 1984; Rognum *et al.*, 1987*b*), but in pathological conditions – inflammation, dysplasia, carcinoma – the epithelium may express varying amounts of these antigens (Rognum *et al.*, 1983; 1987*b*; Ruiz-Cabello *et al.*, 1988).

Möller *et al.* (1991) evaluated expression of HLA-A, B, C, and HLA-DR molecules, invariate chain, and LFA-3 (CD 58) in colorectal carcinoma, and their impact on tumour recurrence. The follow-up ranged from 65 to 45 months. They found that an induction of HLA-DR molecules was seen in 55% of the tumours, but the presence was not correlated with the recurrence rate. However, the authors

**Table IV** Relative risk RR (proportional hazard) with 95% confidence interval (95% CI) for death from colo-rectal cancer according to different risk factors

| *Variable* | *RR* | *95% CI* |
|---|---|---|
| Adjusted for age and sex: | | |
| HLA-DR weak expression *vs* none | 0.63 | (0.36–1.09) |
| HLA-DR strong expression *vs* none | 0.16 | (0.04–0.65) |
| Adjusted[a] | | |
| HLA-DR weak expression *vs* none | 0.80 | (0.40–1.59) |
| HLA-DR strong expression *vs* none | 0.12 | (0.02–0.61) |
| Dukes' stage D *vs* A | 35.37 | (10.71–116.81) |
| Dukes' stage C *vs* A | 7.03 | (2.63–18.82) |
| Dukes' stage B *vs* A | 1.69 | (0.64–4.47) |
| Rectum *vs* colon | 1.89 | (1.03–3.49) |
| Diploid *vs* aneuploid | 0.80 | (0.40–1.62) |
| Pre-operative CEA pr. 10 $\mu g\ l^{-1}$ | 1.19 | (1.05–1.33) |
| Histological grade, poorly differentiated *vs* the rest | 1.13 | (0.48–2.67) |

[a]Estimates of relative risk adjusted for age, sex, and mutually for other variables in the table.

**Figure 4** Survival curves (corrected for death not due to cancer) for patients whose tumours have no HLA-DR expression (——); weak DR expression, 0.1–1.0 (.....); and strong DR expression, >1.0 (---); according to Dukes' stages. Stratified *P* value is less than 0.001.

based their calculations on recurrences and not survival time, and they only judged the immunohistochemical staining to be either positive or negative. Furthermore, they applied a standard indirect immunoperoxidase method, whereas we used an indirect immunofluorescence technique. The latter method may perhaps make it easier to distinguish between different degrees of staining intensity, though the advantages of immunofluorescence techniques over immunoperoxidase has not been proven in quantitative evaluation of antigens in artificial tissue blocks (Valnes *et al.*, 1984). We are now comparing the alkaline phosphatase anti-alkaline phosphatase (APAAP) technique with the immunofluorescence method in a blind study.

In the present study, with a follow-up period of 10–14 years, 52% of the tumours showed positive staining for HLA-DR antigens in varying degrees. This is in agreement with previous studies which report detectable staining in about half of all colon carcinomas (Gutierrez *et al.*, 1987; Degener *et al.*, 1988; Ruiz-Cabello *et al.*, 1988; Möller *et al.*, 1991).

In 39% of the tumours heterogenous staining was found, which is in accordance with Daar & Fabre (1983). Heterogenous staining of HLA-DR in large bowel carcinomas has been claimed to be more common in well differentiated tumours, while the antigen expression becomes more homogeneous with decreasing degrees of differentiation (Rognum *et al.*, 1983). This would be in agreement with the clonal proliferation theory of tumour development proposed by Nowell (1976). In the present study we could not find any relationship between heterogeneity and Dukes' stage. However, poorly differentiated tumours tend to be more often DR-positive and show a more homogeneous staining pattern. This finding in part confirms our previous suggestions (Rognum *et al.*, 1983).

Because of the significant prognostic impact, we think that staining for HLA-DR can profitably be included in the routine diagnostic procedure. Though the operation of scoring is a subjective exercise, our procedure showed good inter- and intraobserver reproducibility. Patients with negative tumours should then be followed up more closely and perhaps receive adjuvant therapy.

Our findings give support to the possibility of immunomodulation of tumours. The presence of HLA-DR on the surface membrane of the tumour cells is a prerequisite for activation of the CD 4+ T-cells and thereby release of cytokines, such as tumour necrosis factor-alpha (TNF) and γ-interferon (INF). Induction of these determinants in clinically manifest carcinomas might help the patients' own immune system to inhibit further tumour growth. A human colonic carcinoma cell line which spontaneously synthesises surface HLA-DR has *in vitro* been stimulated to synthesise surface HLA-DR up to 19-fold when incubated in lymphocyte conditioned medium or with recombinant γ-interferon (Lampert *et al.*, 1985). In another colonic carcinoma cell line TNF markedly and synergistically augmented INF-induced *de novo* synthesis of HLA-DR molecules (Kvale *et al.*, 1988), but TNF alone did not induce detectable levels of DR surface molecules. However, since treatment with TNF has failed to be of any effect in cancer patients (Rosenberg *et al.*, 1989), transfection of the TNF gene into tumour-infiltrating lymphocytes may turn out to be a better approach also in humans (Rosenberg, 1992). Another possibility might be manipulation of the tumour cells by gene therapy. Enhanced expression of class II molecules on tumour cells can thus be achieved by transfected cytokine genes or by direct MHC gene transfection (James *et al.*, 1991). Recently a new gene therapy trial for malignant melanoma, injecting gene copies encased in liposomes into the tumour, has been commented on (Hoffman, 1992). Our finding may suggest a similar approach in large bowel carcinoma by injection of gene copies into the tumour, encoding HLA-DR determinants which, displayed on the tumour cell surface, might trigger an immune response.

The corresponding author is a Research Fellow of the Norwegian Cancer Society. The study was also supported by Torsted's legacy.

The authors wish to thank Hanne Malmström and Anne Gunn Winge for skilled assistance. We are also indebted to Liv Lie from the Cancer Registry of Norway for helping to trace the patients.

### References

ASHLEY, J.B. (1978). *Evans' Histological Appearance of Tumours*, Pp. 582–587. Livingstone: Edinburgh.

BRANDTZAEG, P. (1974). Mucosal and glandular distribution of immunoglobulin components. Immunohistochemistry with a cold ethanol-fixation technique. *Immunology*, **26**, 1101–1114.

BRANDTZAEG, P. & ROGNUM, T.O. (1983). Evaluation of tissue preparation methods and paired immunofluorescence staining for immunocytochemistry of lymphomas. *Histochem. J.*, **15**, 655–689.

CONCHA, A., ESTEBAN, F., CABRERA, T., RUIZ-CABELLO, F. & GARRIDO, F. (1991). Tumor aggressiveness and MHC class I and II antigens in laryngeal and breast cancer. *Cancer Biol.*, **2**, 47–54.

COX, D. (1972). Regression models and life tables. *J. Royal Stat. Soc. (B)*, **34**, 187–200.

DAAR, A.S., FUGGLE, S.V., TING, A. & FABRE, J.W. (1982). Anomalous expression of HLA-DR antigens on human colorectal cancer cells. *J. Immunol.*, **129**, 447–449.

DAAR, A.S. & FABRE, J.W. (1983). The membrane antigens of human colorectal cancer cells: Demonstration with monoclonal antibodies of heterogeneity within and between tumours and of anomalous expression of HLA-DR. *Eur. J. Cancer Clin. Oncol.*, **19**, 209–220.

DAAR, A.S., FUGGLE, S.V., FABRE, J.W., TING, A. & MORRIS, P.J. (1984). The detailed distribution of MHC class II antigens in normal human organs. *Transplantation*, **38**, 293–298.

DEGENER, T., MOMBURG, F. & MÖLLER, P. (1988). Differential expression of HLA-DR, HLA-DP, HLA-DQ and associated invariant chain (Ii) in normal colorectal mucosa, adenoma and carcinoma. *Virchows Archiv A*, **412**, 315–322.

DUKES, C.E. & BUSSEY, H.J.R. (1958). The spread of rectal cancer and its effect on prognosis. *Br. J. Cancer*, **12**, 309–320.

ESTEBAN, F., RUIZ-CABELLO, F., CONCHA, A., PÉREZ-AYALA, M., SÁNCHEZ-ROZAS, J.A. & GARRIDO, F. (1990). HLA-DR expression is associated with excellent prognosis in squamous cell carcinoma of the larynx. *Clin. Exp. Metastasis*, **8**, 319–328.

GHOSH, A.K., MOORE, M., STREET, A.J., HOWAT, J.M.T. & SCHOFIELD, P.F. (1986). Expression of HLA-D subregion products on human colorectal carcinoma. *Int. J. Cancer*, **38**, 459–464.

GUTIERREZ, J., LÓPEZ-NEVOT, M.A., CABRERA, T., OLIVA, R., ESQUIVIAS, J., RUIZ-CABELLO, F. & GARRIDO, F. (1987). Class I and II HLA antigen distribution in normal mucosa, adenoma and colon carcinoma: relation with malignancy and invasiveness. *Expl. Clin. Immunogenet.*, **4**, 144–152.

HOFFMAN, M. (1992). Gene therapy. New clinical trial planned. *Science*, **256**, 305.

JAMES, R.F.L., EDWARDS, S., HUI, K.M., BASSETT, P.D. & GROSVELD, F. (1991). The effect of class II gene transfection on the tumourigenicity of the H-2K-negative mouse leukaemia cell line K36.16. *Immunology*, **72**, 213–218.

JASS, J.R., ATKIN, W.S., CUZICK, J., BUSSEY, H.J.R., MORSON, B.C., NORTHOVER, J.M.A., TODD, I.P. (1986). The grading of rectal cancer: Historical perspectives and a multivariate analysis of 447 cases. *Histopathology*, **10**, 437–459.

KVALE, D., BRANDTZAEG, P. & LØVHAUG, D. (1988). Up-regulation of the expression of secretory component and HLA molecules in a human colonic cell line by tumour necrosis factor-α and gamma interferon. *Scand. J. Immunol.*, **28**, 351–357.

LAMPERT, I.A., KIRKLAND, S., FARRELL, S. & BORYSIEWICZ, L.K. (1985). HLA-DR expression in a human colonic carcinoma cell line. *J. Pathol.*, **146**, 337–344.

LANDIS, J.R. & KOCH, G.G. (1977). The measurement of observer agreement for categorical data. *Biometrics*, **33**, 159–174.

MELING, G.I., ROGNUM, T.O., CLAUSEN, O.P.F., BØRMER, O, LUNDE, O.C., SCHLICHTING, E., GRÜNER, O.P., HOGNESTAD, J., TRONDSEN, E., HAVIG, Ø. & BERGAN, A. (1992). Serum carcinoembryonic antigen in relation to survival, DNA ploidy pattern, and recurrent disease in 406 colorectal carcinoma patients. *Scand. J. Gastroenterol.*, **27**, 1061-1068.

MOLLNES, T.E., LEA, T., HARBOE, M. & TSCHOPP, J. (1985). Monoclonal antibodies recognizing a neoantigen of poly(C9) detect the human terminal complement complex in tissue and plasma. *Scand. J. Immunol.*, **22**, 183-195.

MÖLLER, P., KORETZ, K., SCHLAG, P. & MOMBURG, F. (1991). Frequency of abnormal expression of HLA-A, B, C and HLA-DR molecules, invariant chain, and LFA-3 (CD58) in colorectal carcinoma and its impact on tumor recurrence. *Int. J. Cancer: Suppl.*, **6**, 155-162.

NOWELL, P.C. (1976). The clonal evolution of tumour cell populations. Acquired genetic lability permits stepwise selection of variant sublines and underlies tumor progression. *Science*, **194**, 23-28.

REMMELE, W. & HEINE, M. (1981). Pathologisch-anatomische Begutachtung Kolorektaler Karzinome unter prognostischen Gesichtspunkten. *Med. Welt*, **32**, 771-777.

ROGNUM, T.O., BRANDTZAEG, P. & THORUD, E. (1983). Is heterogenous expression of HLA-DR antigens and CEA along with DNA-profile variations evidence of phenotypic instability and clonal proliferation in human large bowel carcinomas? *Br. J. Cancer*, **48**, 543-551.

ROGNUM, T.O. (1986). A new approach in carcinoembryonic antigen-guided follow-up of large bowel carcinoma patients. *Scand. J. Gastroenterol.*, **21**, 641-649.

ROGNUM, T.O., THORUD, E. & LUND, E. (1987*a*). Survival of large bowel carcinoma patients with different DNA ploidy. *Br. J. Cancer*, **56**, 633-636.

ROGNUM, T.O., BRANDTZAEG, P., ELGJO, K. & FAUSA, O. (1987*b*). Heterogenous epithelial expression of class II (HLA-DR) determinants and secretory component related to dysplasia in ulcerative colitis. *Br. J. Cancer*, **56**, 419-424.

ROGNUM, T.O., LUND, E., MELING, G.I. & LANGMARK, F. (1991). Near diploid large bowel carcinomas have better five-year survival than aneuploid ones. *Cancer*, **68**, 1077-1081.

ROSENBERG, S.A., LOTZE, M.T., YANG, J.C., AEBERSOLD, P.M., LINEHAN, W.M., SEIPP, C.A., & WHITE, D.E. (1989). Experience with the use of high-dose interleukin-2 in the treatment of 652 cancer patients. *Ann. Surg.*, **210**, 474-485.

ROSENBERG, S.A. (1992). The immuntherapy and gene therapy of cancer. *J. Clin. Oncol.*, **10**, 180-199.

RUIZ-CABELLO, F., LOPEZ NEVOT, M.A. & GARRIDO, F. (1988). MHC class I and II gene expression on human tumors. *Adv. Exp. Med. Biol.*, **233**, 119-128.

STAVE, R. & BRANDTZAEG, P. (1977). Fluorescence staining of gastric mucosa. A study with special reference to parietal cells. *Scand. J. Gastroenterol.*, **12**, 885-891.

TURNBULL, R.B., KYLE, K., WATSON, F.R. & SPRATT, J. (1967). Cancer of the colon: the influence of the no-touch isolation technique on survival rates. *Ann. Surg.*, **166**, 420-427.

VALNES, K., BRANDTZAEG, P. & ROGNUM, T.O. (1984). Sensitivity and efficiency of four immunohistochemical methods as defined by staining of artificial sections. *Histochemistry*, **81**, 313-319.

WANEBO, H.J., RAO, B., PINSKY, C.M., HOFFMAN, R.G., STEARNS, M., SCHWARTZ, M.K. & OETTGEN, H.F. (1978). Preoperative carcinoembryonic antigen level as a prognostic indicator in colorectal cancer. *N. Engl. J. Med.*, **299**, 448-451.

WOLLEY, R.C., SCHREIBER, K., KOSS, L.G., KARAS, M. & SHERMAN, A. (1982). DNA distribution in human colon carcinomas and its relationship to clinical behavior. *J. Natl Cancer Inst.*, **69**, 15-22.

ZALOUDIK, J., MOORE, M., GHOSH, A.K., MECHL, Z. & REJTHAR, A. (1988). DNA content and MHC-class-II antigen expression in malignant melanoma: clinical course. *J. Clin. Pathol.*, **41**, 1078-1084.


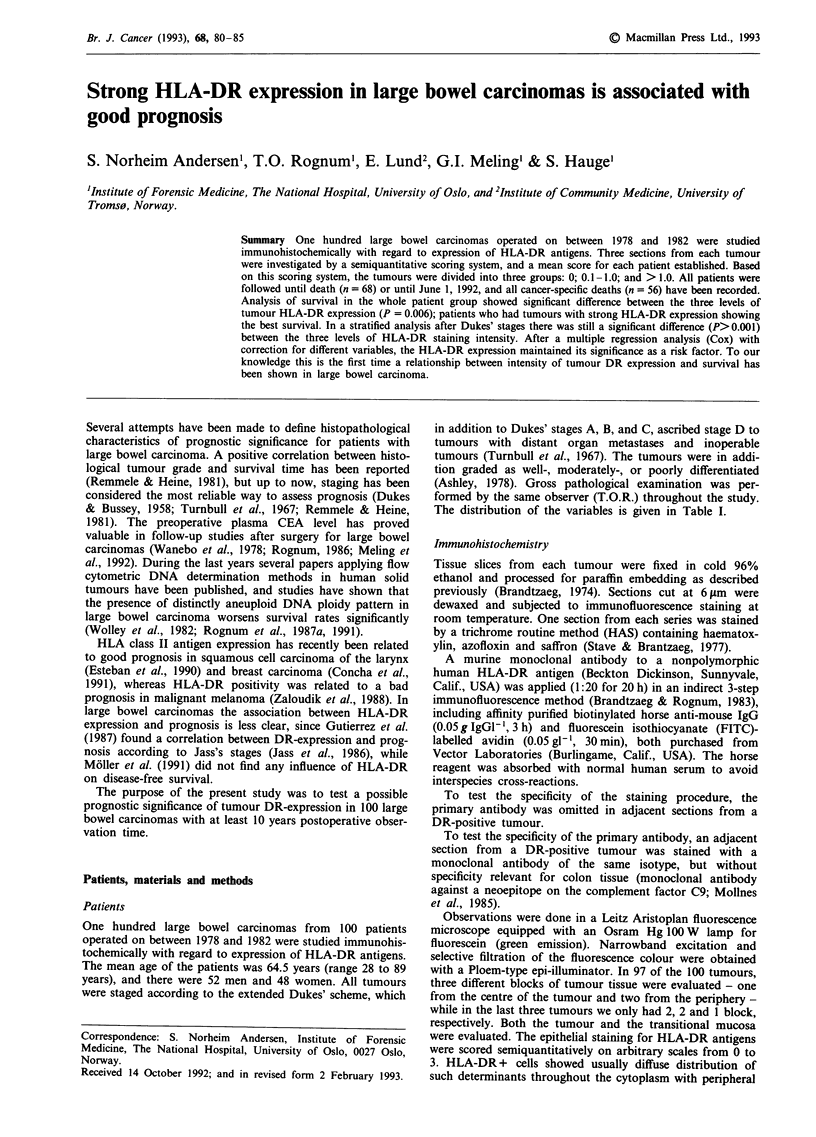

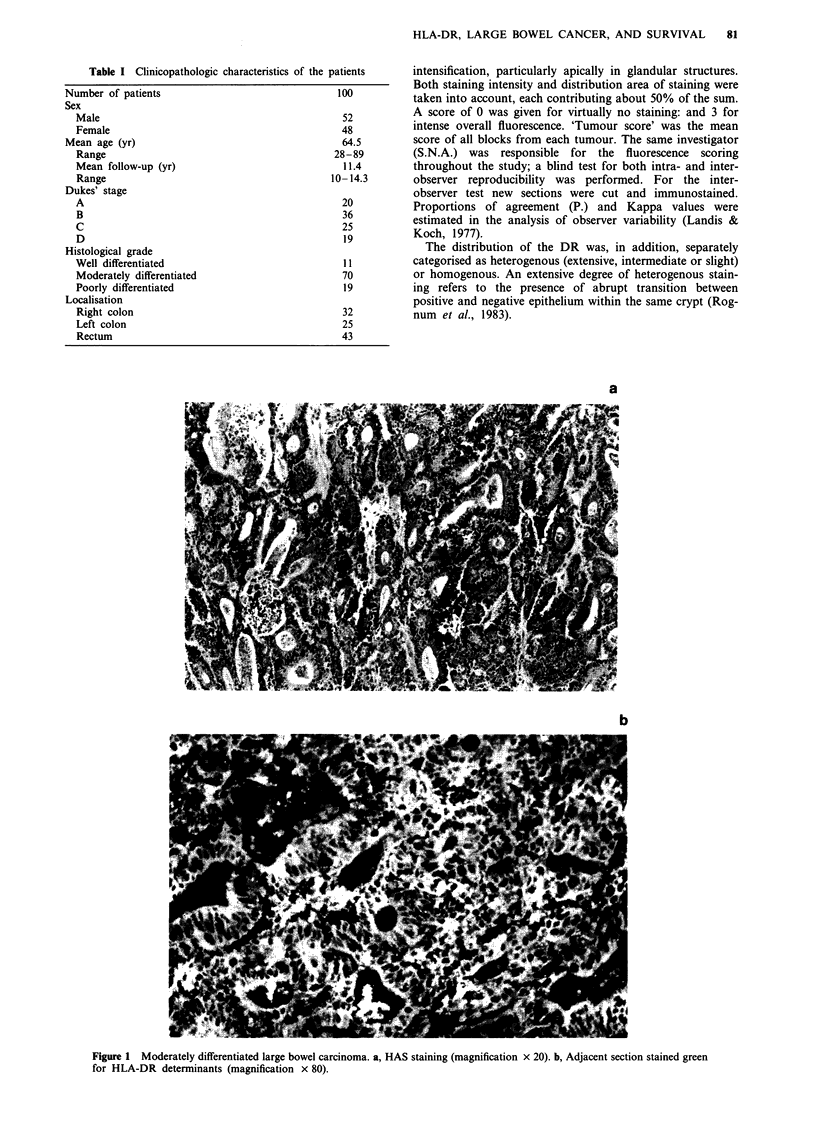

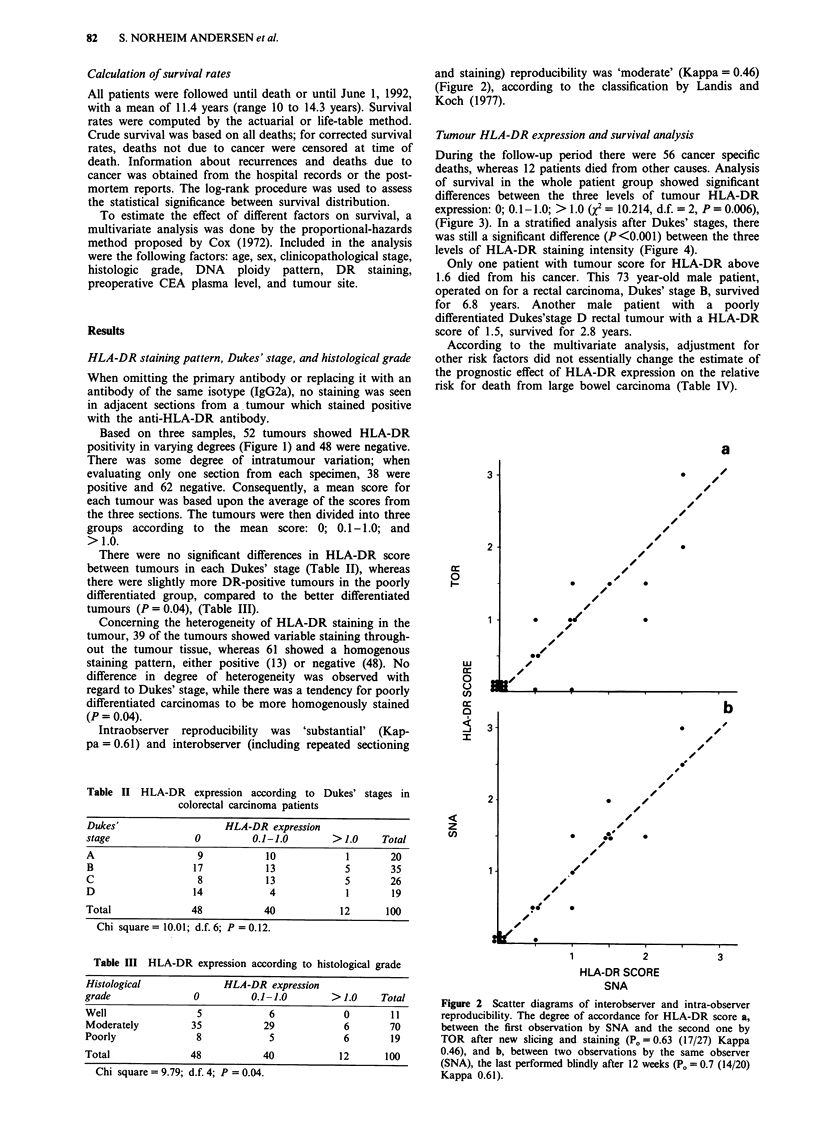

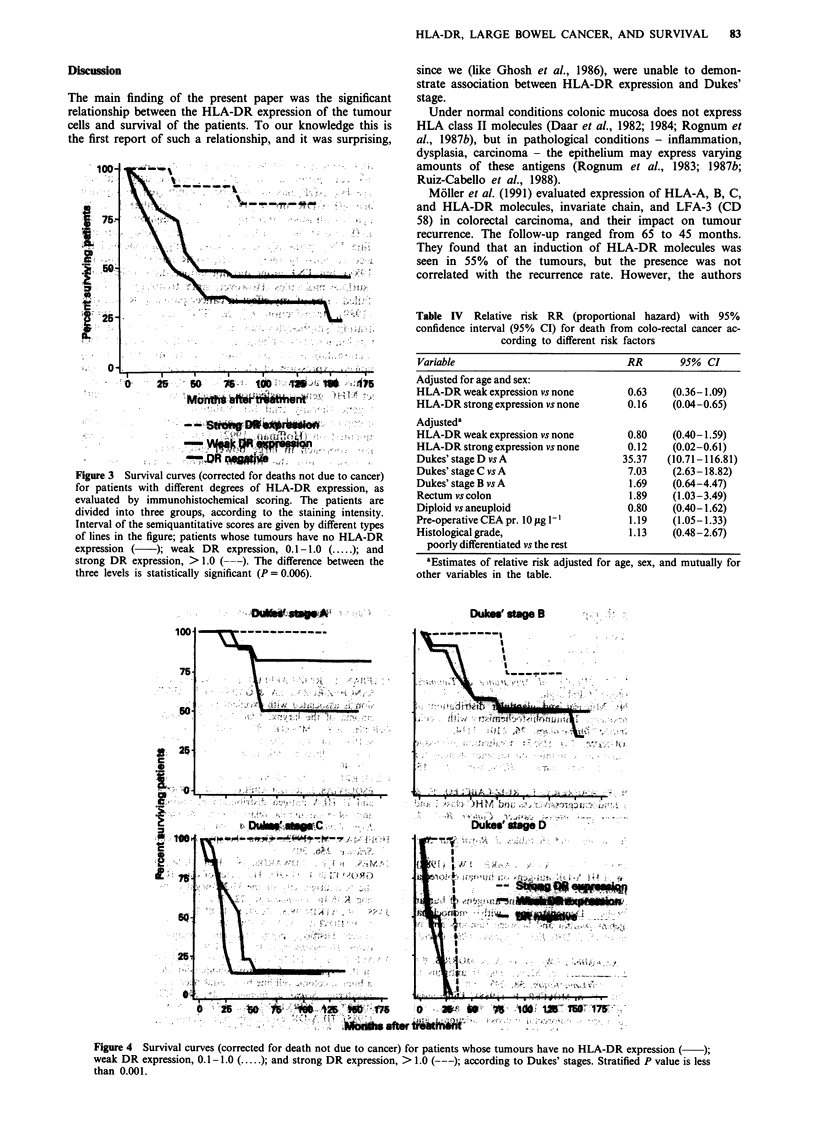

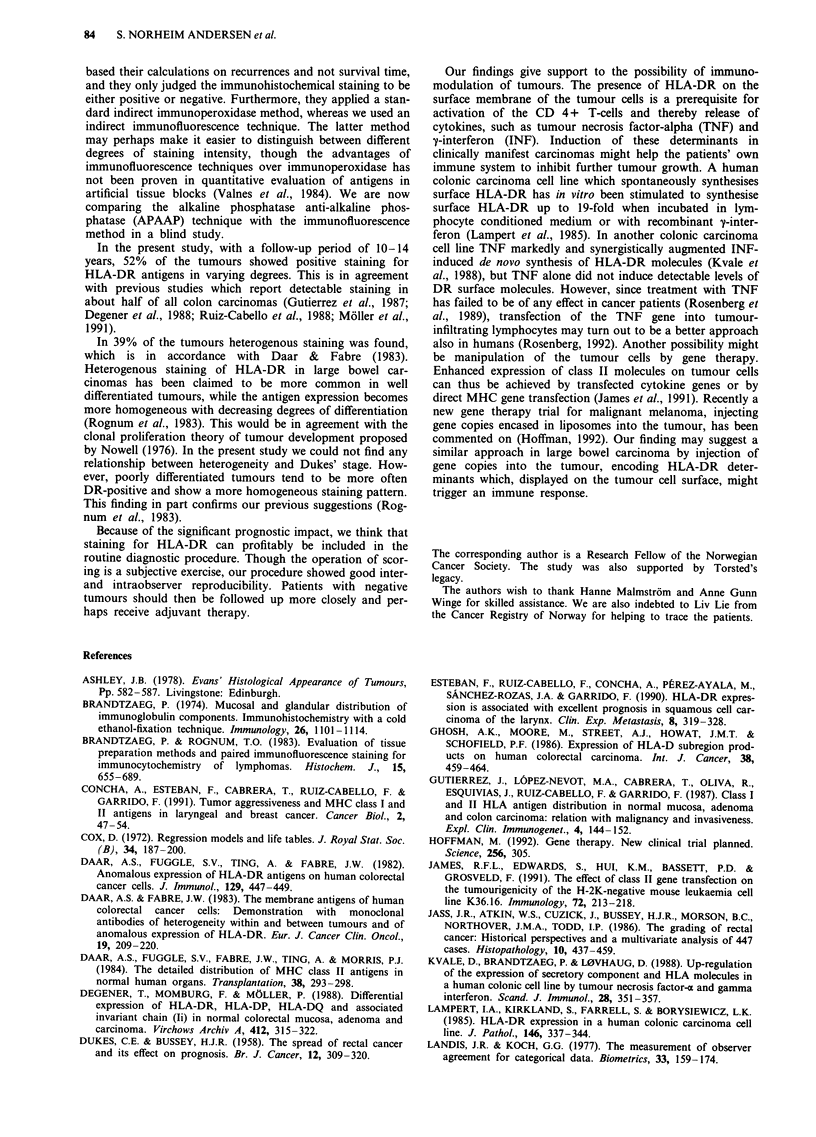

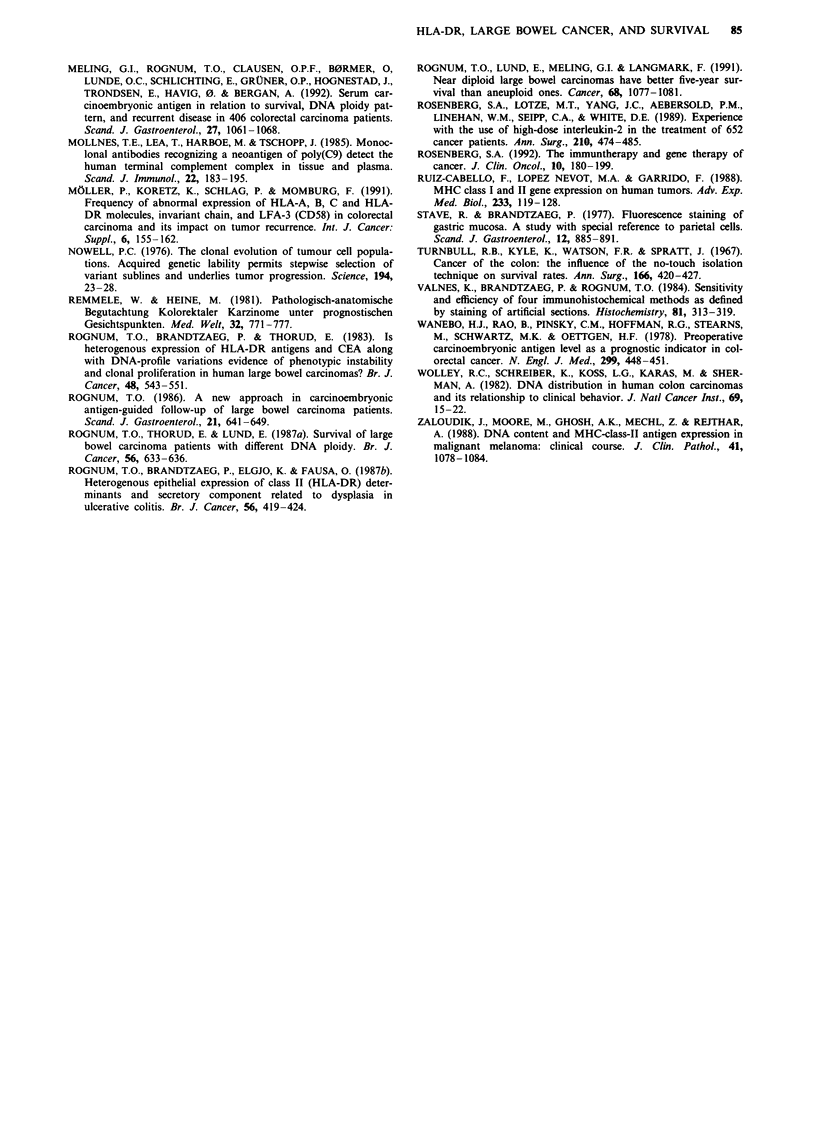


## References

[OCR_00619] Brandtzaeg P. (1974). Mucosal and glandular distribution of immunoglobulin components. Immunohistochemistry with a cold ethanol-fixation technique.. Immunology.

[OCR_00624] Brandtzaeg P., Rognum T. O. (1983). Evaluation of tissue preparation methods and paired immunofluorescence staining for immunocytochemistry of lymphomas.. Histochem J.

[OCR_00630] Concha A., Esteban F., Cabrera T., Ruiz-Cabello F., Garrido F. (1991). Tumor aggressiveness and MHC class I and II antigens in laryngeal and breast cancer.. Semin Cancer Biol.

[OCR_00663] DUKES C. E., BUSSEY H. J. (1958). The spread of rectal cancer and its effect on prognosis.. Br J Cancer.

[OCR_00645] Daar A. S., Fabre J. W. (1983). The membrane antigens of human colorectal cancer cells: demonstration with monoclonal antibodies of heterogeneity within and between tumours and of anomalous expression of HLA-DR.. Eur J Cancer Clin Oncol.

[OCR_00652] Daar A. S., Fuggle S. V., Fabre J. W., Ting A., Morris P. J. (1984). The detailed distribution of MHC Class II antigens in normal human organs.. Transplantation.

[OCR_00640] Daar A. S., Fuggle S. V., Ting A., Fabre J. W. (1982). Anomolous expression of HLA-DR antigens on human colorectal cancer cells.. J Immunol.

[OCR_00657] Degener T., Momburg F., Möller P. (1988). Differential expression of HLA-DR, HLA-DP, HLA-DQ and associated invariant chain (Ii) in normal colorectal mucosa, adenoma and carcinoma.. Virchows Arch A Pathol Anat Histopathol.

[OCR_00667] Esteban F., Ruiz-Cabello F., Concha A., Pérez-Ayala M., Sánchez-Rozas J. A., Garrido F. (1990). HLA-DR expression is associated with excellent prognosis in squamous cell carcinoma of the larynx.. Clin Exp Metastasis.

[OCR_00673] Ghosh A. K., Moore M., Street A. J., Howat J. M., Schofield P. F. (1986). Expression of HLA-D sub-region products on human colorectal carcinoma.. Int J Cancer.

[OCR_00679] Gutierrez J., López-Nevot M. A., Cabrera T., Oliva R., Esquivias J., Ruiz-Cabello F., Garrido F. (1987). Class I and II HLA antigen distribution in normal mucosa, adenoma and colon carcinoma: relation with malignancy and invasiveness.. Exp Clin Immunogenet.

[OCR_00686] Hoffman M. (1992). New clinical trial planned.. Science.

[OCR_00690] James R. F., Edwards S., Hui K. M., Bassett P. D., Grosveld F. (1991). The effect of class II gene transfection on the tumourigenicity of the H-2K-negative mouse leukaemia cell line K36.16.. Immunology.

[OCR_00696] Jass J. R., Atkin W. S., Cuzick J., Bussey H. J., Morson B. C., Northover J. M., Todd I. P. (1986). The grading of rectal cancer: historical perspectives and a multivariate analysis of 447 cases.. Histopathology.

[OCR_00702] Kvale D., Brandtzaeg P., Løvhaug D. (1988). Up-regulation of the expression of secretory component and HLA molecules in a human colonic cell line by tumour necrosis factor-alpha and gamma interferon.. Scand J Immunol.

[OCR_00708] Lampert I. A., Kirkland S., Farrell S., Borysiewicz L. K. (1985). HLA-DR expression in a human colonic carcinoma cell line.. J Pathol.

[OCR_00713] Landis J. R., Koch G. G. (1977). The measurement of observer agreement for categorical data.. Biometrics.

[OCR_00719] Meling G. I., Rognum T. O., Clausen O. P., Børmer O., Lunde O. C., Schlichting E., Grüner O. P., Hognestad J., Trondsen E., Havig O. (1992). Serum carcinoembryonic antigen in relation to survival, DNA ploidy pattern, and recurrent disease in 406 colorectal carcinoma patients.. Scand J Gastroenterol.

[OCR_00727] Mollnes T. E., Lea T., Harboe M., Tschopp J. (1985). Monoclonal antibodies recognizing a neoantigen of poly(C9) detect the human terminal complement complex in tissue and plasma.. Scand J Immunol.

[OCR_00733] Möller P., Koretz K., Schlag P., Momburg F. (1991). Frequency of abnormal expression of HLA-A,B,C and HLA-DR molecules, invariant chain, and LFA-3 (CD58) in colorectal carcinoma and its impact on tumor recurrence.. Int J Cancer Suppl.

[OCR_00740] Nowell P. C. (1976). The clonal evolution of tumor cell populations.. Science.

[OCR_00746] Remmele W., Heine M. (1981). Pathologisch-anatomische Begutachtung kolorektaler Karzinome unter prognostischen Gesichtspunkten.. Med Welt.

[OCR_00758] Rognum T. O. (1986). A new approach in carcinoembryonic antigen-guided follow-up of large-bowel carcinoma patients.. Scand J Gastroenterol.

[OCR_00768] Rognum T. O., Brandtzaeg P., Elgjo K., Fausa O. (1987). Heterogeneous epithelial expression of class II (HLA-DR) determinants and secretory component related to dysplasia in ulcerative colitis.. Br J Cancer.

[OCR_00751] Rognum T. O., Brandtzaeg P., Thorud E. (1983). Is heterogeneous expression of HLA-dr antigens and CEA along with DNA-profile variations evidence of phenotypic instability and clonal proliferation in human large bowel carcinomas?. Br J Cancer.

[OCR_00774] Rognum T. O., Lund E., Meling G. I., Langmark F. (1991). Near diploid large bowel carcinomas have better five-year survival than aneuploid ones.. Cancer.

[OCR_00763] Rognum T. O., Thorud E., Lund E. (1987). Survival of large bowel carcinoma patients with different DNA ploidy.. Br J Cancer.

[OCR_00785] Rosenberg S. A. (1992). Karnofsky Memorial Lecture. The immunotherapy and gene therapy of cancer.. J Clin Oncol.

[OCR_00779] Rosenberg S. A., Lotze M. T., Yang J. C., Aebersold P. M., Linehan W. M., Seipp C. A., White D. E. (1989). Experience with the use of high-dose interleukin-2 in the treatment of 652 cancer patients.. Ann Surg.

[OCR_00789] Ruiz-Cabello F., Nevot M. A., Garrido F. (1988). MHC class I and II gene expression on human tumors.. Adv Exp Med Biol.

[OCR_00794] Stave R., Brandtzaeg P. (1977). Fluorescence staining of gastric mucosa. A study with special reference to parietal cells.. Scand J Gastroenterol.

[OCR_00799] Turnbull R. B., Kyle K., Watson F. R., Spratt J. (1967). Cancer of the colon: the influence of the no-touch isolation technic on survival rates.. Ann Surg.

[OCR_00804] Valnes K., Brandtzaeg P., Rognum T. O. (1984). Sensitivity and efficiency of four immunohistochemical methods as defined by staining of artificial sections.. Histochemistry.

[OCR_00809] Wanebo H. J., Rao B., Pinsky C. M., Hoffman R. G., Stearns M., Schwartz M. K., Oettgen H. F. (1978). Preoperative carcinoembryonic antigen level as a prognostic indicator in colorectal cancer.. N Engl J Med.

[OCR_00817] Wolley R. C., Schreiber K., Koss L. G., Karas M., Sherman A. (1982). DNA distribution in human colon carcinomas and its relationship to clinical behavior.. J Natl Cancer Inst.

[OCR_00821] Zaloudik J., Moore M., Ghosh A. K., Mechl Z., Rejthar A. (1988). DNA content and MHC class II antigen expression in malignant melanoma: clinical course.. J Clin Pathol.

